# Phase II clinical trial of sequential treatment with systemic chemotherapy and intraperitoneal paclitaxel for gastric and gastroesophageal junction peritoneal carcinomatosis - STOPGAP trial

**DOI:** 10.1186/s12885-023-10680-1

**Published:** 2023-03-04

**Authors:** Maheswari Senthil, Farshid Dayyani

**Affiliations:** 1 Division of Surgical Oncology, University of California Irvine, 3800 Chapman Ave, Ste 6400, CA 92868 Orange, USA; 2 Division of Hematology Oncology, University of California Irvine, CA Orange, USA

**Keywords:** Gastric cancer, Peritoneal carcinomatosis, GEJ adenocarcinoma, Intraperitoneal chemotherapy, Paclitaxel

## Abstract

**Background:**

Studies from Asia indicate that normothermic intraperitoneal chemotherapy (NIPEC) may confer survival benefit in patients with gastric peritoneal carcinomatosis (PC). However, data regarding this approach is lacking in western population. The current STOPGAP trial is intended to assess 1-year progression-free survival benefit of sequential systemic chemotherapy and paclitaxel NIPEC in patients with gastric/ gastroesophageal junction (GEJ) adenocarcinoma PC.

**Methods:**

This is a prospective, single center, single arm, phase II investigator-initiated clinical trial. Patients with histologically proven gastric/GEJ (Siewert 3) adenocarcinoma with positive peritoneal cytology or PC will be eligible to participate after three months of standard of care systemic chemotherapy and with no evidence of visceral metastasis on restaging scans. The primary treatment is iterative paclitaxel NIPEC with systemic paclitaxel and 5-fluorouracil, which will be administered on days1 and 8 and repeated every three weeks for 4 cycles. Patients will undergo diagnostic laparoscopy both before and after NIPEC to assess peritoneal cancer index (PCI). Patients with PCI less than or equal to 10 in whom complete cytoreduction (CRS) is feasible may opt to undergo CRS with heated intraperitoneal chemotherapy (HIPEC). The primary endpoint is 1-year progression free survival and secondary endpoints are overall survival and patient reported quality of life outcomes measured by EuroQol- 5 dimensions-5 level (EuroQol-5D-5L) questionnaire.

**Discussion:**

If the sequential approach of systemic chemotherapy followed by paclitaxel NIPEC proves beneficial, then this approach could be used in larger, muti-institutional randomized clinical trial of gastric PC.

**Trial Registration:**

The trial was registered on 21/02/2021, under clinical trials.gov; Identifier: NCT04762953.

## Background and rationale

Gastric cancer is the fifth most common cancer and fourth leading cause of cancer deaths worldwide in 2020 [1]. In the U.S., in 2022 an estimated 26,380 new gastric cancer cases will be diagnosed and 11,090 people will die due to this cancer [2]. The observed high mortality in gastric cancer is due to the high incidence of metastasis, particularly peritoneal carcinomatosis (PC). Incidence of PC in gastric cancer is about 30% at the time of initial presentation [3–5] and 15–52% at the time of recurrence [3, 6–9]. In most patients, peritoneum is the only site of metastatic disease. The survival for patients with PC is dismal with median survival of 4–7 months [3, 6, 10]. In the FLOT 3 study that tested the efficacy of a triplet regimen consisting of 5-FU, leucovorin and docetaxel in the management of gastric/GEJ adenocarcinoma, the median survival of the group that comprised of majority of patients with PC was 10 months [11]. Systemic chemotherapy alone may not be sufficient to treat peritoneal metastasis as evidence suggests that plasma peritoneal barrier reduces penetrance of chemotherapy in the peritoneal cavity [12]. There is a critical need to develop treatment strategies that incorporate systemic and regional treatment in the management of gastric PC to improve survival outcomes.

Efficacy of normothermic intraperitoneal chemotherapy (NIPEC) combined with systemic chemotherapy to treat gastric cancer PC has been extensively studied in Asia [13–17]. Recent phase III randomized controlled trial, PHOENIX GC from Japan showed that intraperitoneal (IP) and intravenous (IV) paclitaxel with S-1 had a better 3 -year survival rate compared to IV cisplatin and S-1 alone (21.9% vs. 6%) [18]. Although PHOENIX GC trial failed to meet the primary endpoint of overall survival benefit with IP paclitaxel due to multiple reasons including imbalances between the IP and systemic treatment only arm, with more patients with moderate ascites randomized to the IP group and crossover of patients to IP treatment, the possible benefits of IP paclitaxel require further exploration. Regional therapy with IP paclitaxel is a viable strategy to treat PC as paclitaxel, due to its large molecular weight and lipophilic nature is largely retained in the peritoneal cavity, achieving high IP drug levels. A phase I study by Imano et al. that evaluated the safety and efficacy of a single intraperitoneal administration of paclitaxel (80 mg/m2), followed by intravenous administration of paclitaxel (50 mg/m2) plus S-1 (80 mg/m2) a week later, in gastric PC showed that the ratio of (AUC peritoneal)/(AUC plasma) was 1065:1 on pharmacokinetic analysis, indicating that majority of intraperitoneally administered paclitaxel is retained in the peritoneal cavity [13]. Multiple phase I and phase II studies have assessed the optimal dose and safety of IP paclitaxel with doses ranging from 20 mg/m2 to 80 mg/m2 with reasonable toxicity profile [19–21]. An ongoing phase II clinical trial in the National Cancer Institute in the United States is testing a dose of 60 mg/m2 of IP paclitaxel (NCT04034251). Furthermore, the practical advantages of NIPEC are that it is administered in clinic setting and can be repeated several times based on response. In the PHOENIX GC trial, the median duration of treatment for patients in the IP arm was 39 weeks, which is approximately 13 cycles of IP chemotherapy [18].

Due to the inherent biologic differences in gastric cancer between eastern and western countries, the treatment strategy of paclitaxel NIPEC with systemic chemotherapy backbone needs to be tested in a clinical trial setting in gastric PC in the western population.*We hypothesize that sequential systemic chemotherapy followed by intraperitoneal paclitaxel is safe and will improve 1-year PFS in gastric/gastroesophageal junction adenocarcinoma (GEJ) (Siewert 3) with peritoneal carcinomatosis compared to historic controls.* The initial systemic therapy provides systemic downstaging, disease control and allows for selection of patients who have not had progression before initiating intraperitoneal therapy. Based on response to therapy and extent of disease, consolidation surgery with Cytoreduction (CRS) with heated intraperitoneal chemotherapy (HIPEC) may be beneficial in the appropriately selected patients. A retrospective study ( CYTO CHIP) that evaluated the long-term outcomes after CRS/HIPEC reported a 5-year survival rate of 18%, and the factors associated with improved survival were low peritoneal cancer index (PCI) and complete cytoreduction ( CC-0) [22]. In this study, all patients who were disease free after 5 years had PCI ≤ 7 and underwent CC-0 [22]. Currently, PERISCOPE II, a phase III randomized controlled trial is underway to evaluate the survival benefits of CRS/HIPEC combined with systemic chemotherapy in gastric PC patients with PCI ≤ 7 as compared to systemic chemotherapy alone [23]. In addition to possible disease control and survival benefit, the other advantages of IP therapy are palliation of symptomatic ascites and use of IP port to drain ascites which would improve quality of life outcomes.

Long-term survivors with stage IV gastric PC after CRS/HIPEC are well described. However, all randomized available data to date have failed to consistently show a survival benefit in gastric PC using IP chemotherapy and/or CRS/HIPEC in unselected all comer populations. Hence, this is likely due to lack of appropriate patient selection, and possibly also due to a lack of adequate conversion therapy in the induction phase. If there is strong signal favoring paclitaxel NIPEC therapy, this approach has the potential to be considered as one of the treatment arms in a future large, randomized, multi-institutional trial.

## Methods

### Study design

This is a single-center, single arm, phase II clinical trial for patients with histologically proven gastric or gastroesophageal junction (GEJ) (Siewert 3) adenocarcinoma with positive peritoneal cytology or PC. The primary treatment is iterative paclitaxel NIPEC with systemic paclitaxel and 5-fluorouracil after three months of standard of care systemic chemotherapy. The ongoing study is approved by the institutional review board of University of California Irvine (UCI) [HS#2020–6178] and is supported by the Stern Center Clinical Trials Office at UCI and an UCI Cancer Center anti-cancer challenge grant. The trial was registered under clinical trials.gov; Identifier: NCT04762953 on 21/02/2021. All study methods are performed in accordance with the relevant guidelines and regulations (Declaration of Helsinki).

### Study population

**Fig. 1:**
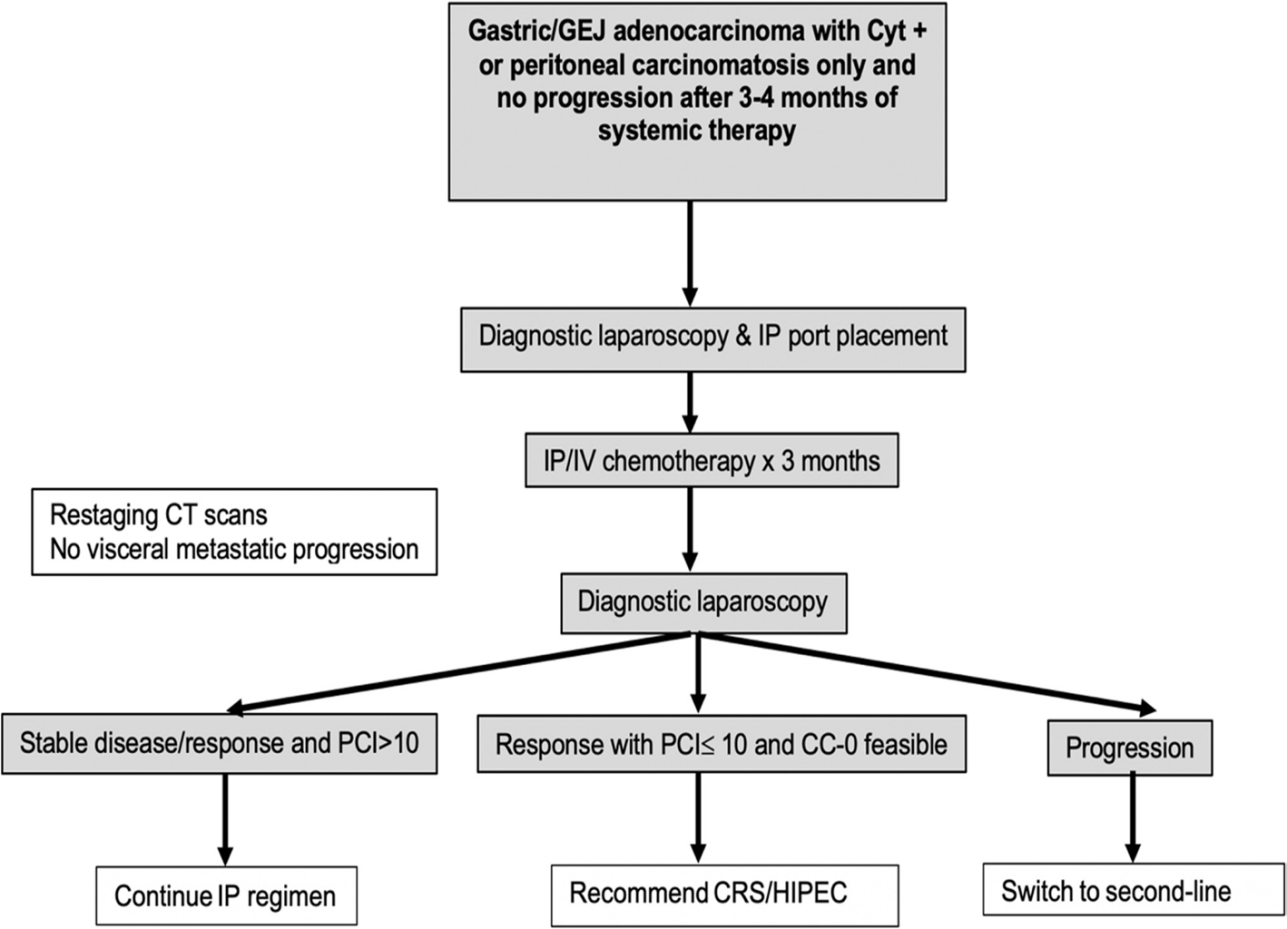
Study schema

Adult patients (18–75 years old) with gastric and GEJ adenocarcinoma with positive peritoneal cytology or peritoneal carcinomatosis detected by laparoscopy, laparotomy, or imaging and without evidence of distant organ metastasis and no evidence of progression after 3–4 months of first-line systemic therapy are eligible for this study (Fig. 1 Study Schema). Systemic therapy prior to enrollment will be at the discretion of the treating physician including the use of PD-1/PD-L1 and HER-2 blockade based on tumor analysis. Informed consent will be obtained from all study participants.

The key inclusion and exclusion criteria are listed in Table 1.

**Table 1:** STOPGAP trial key inclusion and exclusion criteria

Inclusion criteria	Exclusion criteria
Patients (age ≥ 18 -75 years) must have treatment naïve histologically or cytologically confirmed gastric or gastroesophageal adenocarcinoma and have received 3–4 months of first line systemic treatment without visceral metastatic progression	Any evidence of extensive retroperitoneal lymph node metastases not amenable to resection during gastrectomy
Must have peritoneal cytology positive disease or peritoneal carcinomatosis detected by imaging, laparoscopy, or laparotomy	Any evidence of small or large bowel obstruction except for gastric outlet obstruction due to primary malignancy
Performance status: ECOG performance status ≤ 2. ECOG 2 allowed if attributed to malignancy (rather than comorbidities)	History of another primary cancer within the last 3 years except for non-melanoma skin cancer, early-stage prostate cancer, or curatively treated cervical carcinoma in-situ and not treated with systemic therapy
Life expectancy of greater than 6 months	Prior surgery that would preclude safe diagnostic laparoscopy and port placement
Adequate organ and marrow function	

Restaging imaging with CT chest and CT abdomen and pelvis and /or diffusion weighted MRI of abdomen and pelvis with contrast will be obtained after completion of systemic therapy. In the absence of distant organ metastatic progression, patients will be deemed eligible to participation in the trial.

### Study procedures

After enrollment, patients will undergo diagnostic laparoscopy, peritoneal washings, evaluation of PCI and biopsies if deemed necessary, and IP port placement (14.3 fr BARD IP port). PCI is a well-established scoring system described by Sugarbaker et al. that is used to assess and document the extent of peritoneal disease [24]. For this purpose, the peritoneal cavity is divided in 13 well-defined regions. In each of the 13 regions, the size of the largest tumor nodule is measured and given a score: No tumor-0; < 0.5 cm – 1; 0.5 cm-5 cm -2; > 5 cm or confluent tumor nodules—3. The PCI is calculated by adding the scores of all 13 regions.

#### Treatment

IP regimen consists of IV Paclitaxel, 5- FU and Leucovorin and IP Paclitaxel (Table 2). Although in PHOENIX GC trial the IP dose was 20 mg/m2, given the safety data for higher doses of IP paclitaxel, we chose an IP dose of 40 mg/m2. However, the systemic backbone portion of the regimen used in PHOENIX GC was adopted as such with the substitution of 5-FU and leucovorin instead of S-1 as S-1 is not available in the United States.

**Table 2:** STOPGAP intraperitoneal chemotherapy regimen

Agent	Dose	Route	Schedule
Leucovorin	20 mg/m2	IV	Days 1 and 8
5-FU	400 mg/m2	IV	Days 1 and 8
Paclitaxel	50 mg/m2	IV	Days 1and 8
Paclitaxel	40 mg/m2	IP	Days 1and 8

Paclitaxel 40 mg/m2 in 500 ml of normal saline will be instilled into the peritoneal cavity through the IP port on days 1 and 8, repeated every 21 days for 4 cycles. In patients with moderate ascites, IP port will be used to drain the fluid prior to delivery of IP treatment. Patients with significant worsening of sensory neuropathy from prior systemic treatment may omit IV Paclitaxel in subsequent cycles. Since all enrolled patients have stage IV disease, the addition of nivolumab 360 mg IV on day 1 of each cycle is permitted based on investigator discretion.

Restaging imaging with CT and /or diffusion weighted MRI with contrast is obtained 4–6 weeks after completion of IP chemotherapy. In the absence of progression, patients may undergo diagnostic laparoscopy with biopsies to assess the extent of PCI and treatment response. At this point, based on response, patients will be triaged to one of the following treatment plans: stable disease or response and PCI > 10—continue IP chemotherapy regimen, progression—switch to second line regimen, response with PCI $\le$ 10 and complete cytoreduction is feasible—recommend cytoreduction surgery with HIPEC. Although the CYTOCHIP study showed long term survival benefit was achieved with CRS/HIPEC in patients with PCI ≤ 7, we chose a cutoff of PCI ≤ 10, as the objective of the STOPGAP study is to offer cytoreduction to all eligible patients who have low volume disease in whom a complete cytoreduction is feasible. This is particularly important as the likelihood of prolonged progression free survival without CRS in gastric PC is unlikely. HIPEC is performed using Cisplatin 75 mg/m2 and Mitomycin15 mg/m2 for 90 min at temperature between 41–42 °C. Adjuvant therapy is given based on the discretion of the treating investigator. Routine surveillance with CT scan of the chest, abdomen, and pelvis and/or MRI will be performed every 8–12 weeks. Radiological assessment of disease recurrence will be monitored. Quality of life questionnaire, EQ-5D-5L will be completed by patient every 8 weeks.

#### Endpoints

The primary endpoint is 1-year progression free survival, and the secondary endpoints are overall survival and patient reported quality of life outcomes measured by EQ-5D-5L. Other planned correlative studies include plasma exosomal gene signature associated with response and comparison of plasma exosome NGS analysis with circulating tumor DNA.

### Statistical design and sample size

The sample-size justification is based on the exact, one-sided, binomial test of the primary endpoint, progression free survival at 12 months (viz., PFS12). Based on current evidence, PFS-12 in gastric carcinomatosis with optimal treatment is estimated at less than 10% [12] The hypothesis is that the combination of upfront systemic therapy for 3–4 months followed by IP chemotherapy for 3 months with or without CRS/HIPEC in selected patients is feasible and will improve PFS-12 compared to historical controls. Previous phase II study of iterative HIPEC reported a conversion to surgery rate of 26.3% [25]. We expect that with selection of patients after three months of systemic chemotherapy and iterative NIPEC treatment that the conversion to surgery rate will be 35% in this study. To estimate the efficacy, *n* = 20 patients will be enrolled. If at least *n* = 7 patients have not progressed by 12 months, then the observed PFS-12 of 35% will have a one sided lower 95% CI of 17.5% [26]. This means the lower boundary for estimated efficacy is as good as or better than currently available options in this setting. This test will have at least 80-percent power to reject the null- hypothesis that PFS12 is ten percent in favor of an alternative hypothesis that PFS12 is at least 35 percent with 20 evaluable participants. We seek to enroll 25 subjects to allow for attrition.

## Discussion

Gastric cancer is a global health problem with 1.1 million new cases diagnosed every year, contributing to nearly 800,000 deaths annually, making it the fourth leading cause of cancer related deaths in the world [1]. Nearly 30—40% of patients diagnosed with gastric cancer will present with synchronous PC [8, 10]. Recent studies have shown that systemic chemotherapy alone in the management of gastric carcinomatosis is associated with a dismal survival of 6—15 months [11]. Most patients with gastric PC die due to progression of disease in the peritoneum.

Due to the anarchic circulation of peritoneal metastasis and the plasma peritoneal barrier, the intra tumoral concentrations of systemically administered chemotherapy may not be adequate to achieve desired response [27]. Hence, there is strong clinical rationale to use combination of systemic and regional intraperitoneal therapies in the management gastric PC [28]. Several studies from Asia have been published about the feasibility combining normothermic IP paclitaxel with systemic therapy in gastric PC [21]. Particularly, the PHOENIX GC trial, despite its pitfalls showed that there may be a survival advantage in patients treated with normothermic IP paclitaxel [18]. Despite the widespread acceptance of normothermic intraperitoneal chemotherapy (NIPEC) in Asia for gastric cancer, it has not been tested in the western population. Due to the inherent biologic differences in gastric cancer between east and west, it is important to test this combinatorial approach in western cohort of patients.

The STOPGAP clinical trial is one of two clinical trials in the United States that is testing the role of iterative NIPEC with paclitaxel in gastric PC. In this study, patients with histologically proven gastric/GEJ adenocarcinoma with positive peritoneal cytology or peritoneal carcinomatosis will be enrolled after 3–4 months of optimal multi-agent systemic therapy and no evidence of visceral metastasis. This pragmatic study design allows for patients to get started on systemic treatment without delay and provides a testing period to assess for any visceral metastasis as patients with multiple sites of metastasis will not benefit from this regional approach. Additionally, allowing for upfront systemic therapy will likely induce a tumor response in the primary tumor and contribute to the downstaging of PC prior to IP treatment. We have incorporated laparoscopic assessment of PCI both before and after IP treatment due to the well-known difficulty with accurately assessing PCI using radiologic studies. Peritoneal biopsies will also be obtained to assess pathologic treatment response.

Patients with cytology positive (cyt +) disease without macroscopic evidence of PC as well as patients with macroscopic PC, irrespective of peritoneal disease burden are eligible for this study. We recognize that these are two ends of the spectrum of gastric carcinomatosis. Nevertheless, gastrectomy after neoadjuvant chemotherapy is not considered standard of care for cyt + M1 patients in the United States, even though it is offered to patients in selected centers. Additionally, outcomes of cyt + M1 patients after gastrectomy is worse than M0 patients, hence alternate approaches to improve outcomes are necessary. On the other hand, most patients with gastric PC will present with moderate to high PCI, hence the use of regional therapy combined with systemic therapy to improve survival warrants further evaluation, especially since the current outcomes even with best systemic treatment for this group is dismal. However, it is important to note that even though there is no PCI cut off for enrollment into the IP treatment protocol, cytoreduction will only be offered to patients with PCI ≤ 10 in whom a complete cytoreduction is feasible. Although the PCI cutoff for cytoreduction is somewhat arbitrary, the rationale for selecting this cutoff is based on the general acceptance that PCI less than 10 is considered low volume disease and results from retrospective studies have shown that the PCI of 6 or less is associated with improved survival with CRS. We expect that about 35% of enrolled patients will be eligible for CRS. Nevertheless, the objective of this phase II study is to assess the safety and feasibility of this approach and 1-year progression free survival. We expect that the regional treatment of the peritoneal cavity with NIPEC iterative paclitaxel will offer better disease control. It is important to note that the results of this study is expected to provide evidence for a larger randomized controlled trial with systemic chemotherapy alone as the control arm. The ongoing phase III randomized controlled trial from Netherlands, PERISCOPE II, is comparing systemic chemotherapy alone with systemic chemotherapy with cytoreduction combined with hyperthermic oxaliplatin and normothermic docetaxel in patients with gastric cancer PC with limited peritoneal dissemination (PCI less than 7) and /or positive peritoneal cytology [23]. However, that study is not designed to select surgical candidates based on appropriate response to upfront systemic treatment but help shed light on the role of cytoreduction in gastric PC.

In summary, the study design of STOPGAP is based on several key observations from previous studies; First line multi-agent systemic treatment (i.e., a fluoropyrimidine/platinum-based regimen ± biologics and immune checkpoint inhibitor) has the highest overall response rate in GEC and the maximum response is achieved within the first 3–4 months of systemic treatment. Due to the inherent limitations of systemic chemotherapy in the management of PC, after an initial systemic cytoreduction, a switch to IP chemotherapy can lead to further downstaging of PC without major exacerbation of limiting toxicities (i.e., neuropathy and cytopenia). The total induction period of 6–7 months (including 3 months on STOPGAP) will select for patients with favorable response to chemotherapy. Since complete pathologic response is very unlikely with systemic therapy alone, CRS/HIPEC in appropriately selected patients will be able to remove all residual disease and further decrease the risk of recurrence.

We believe that the STOPGAP clinical trial with iterative paclitaxel NIPEC in the western population is an important next step to expand the role of iterative regional therapies prior to CRS in patients with gastric PC. We also expect that the role of iterative normothermic IP treatment has the potential to be expanded beyond paclitaxel with the new targeted therapies in the pipeline for gastric cancer. Establishing the safety and feasibility of this approach will pave way for testing novel drug combinations that may have significant impact in this deadly disease.

## Data Availability

The datasets used and/or analyzed during the current study can be made available from the corresponding author on reasonable request.
